# A qualitative evaluation of volunteers' experiences in a phase I/II HIV vaccine trial in Tanzania

**DOI:** 10.1186/1471-2334-11-283

**Published:** 2011-10-24

**Authors:** Edith AM Tarimo, Anna Thorson, Thecla W Kohi, Muhammad Bakari, Eric Sandstrom, Fred Mhalu, Asli Kulane

**Affiliations:** 1 Division of Global Health (IHCAR), Department of Public Health Sciences, Karolinska Institutet, Stockholm, Sweden; 2 Department of Nursing Management, Muhimbili University of Health and Allied Sciences, Dar es Salaam, Tanzania; 3 Department of Internal Medicine Muhimbili University of Health and Allied Sciences, Dar es Salaam, Tanzania; 4 Department of Microbiology and Immunology, Muhimbili University of Health and Allied Sciences, Dar es Salaam, Tanzania; 5 Södersjukhuset, Venhalsan, Karolinska Institutet, Stockholm, Sweden

## Abstract

**Background:**

Evaluating experiences of volunteers in an HIV vaccine trial will be useful for the conduct of future trials. The purpose of this study among volunteers who participated in a phase I/II HIV vaccine trial in Dar es Salaam, Tanzania was to assess what characterized their experiences during the trial.

**Methods:**

We conducted four focus group discussions with 35 out of the 60 individuals (women and men) after the five scheduled vaccinations. An interpretive description approach was applied to data analysis.

**Results:**

As a result of the trial interventions, both men and women gained confidence in their own abilities to have safer, less risky sexual behaviour. The participants experienced the trial as a way of accessing free [insured] medical services. Most of the men said they had gone from self-medication to professional medical consultation. Despite these benefits, the participants faced various challenges during the trial. Such challenges included mistrust of the trial shown by health care providers who were not connected to the trial and discouragement from friends, colleagues and family members who questioned the safety of the trial. However, they managed to cope with these doubts by using both personal and trial related interventions.

**Conclusion:**

We found that during the phase I/II HIV vaccine trial, participants had both the opportunities and the ability to cope with the doubts from the surrounding community. Follow up visits enhanced the opportunities and individuals' abilities to cope with the doubts during the trial. Understanding this discourse may be useful for the trial implementers when designing future trials.

**Trials Registration:**

ISRCTN: ISRCTN90053831

Pan African Clinical Trials Registry (PACTR): ATMR2009040001075080

## Background

Participants who enrol in HIV vaccine trials may face diverse problems, including social harm which refers to a negative trial-related experience by a study participant which manifest in psychological, social or physical ways [1]. Previous studies in phases I and II HIV vaccine trials at different sites have shown evidence of trial participants experiencing social harm. These include negative reactions from friends, family, co-workers or disturbance in relationships [2-4]. Also, during the efficacy trials, participants reported concerns by family and friends associated with the perception that volunteers were HIV-infected or were at risk of HIV infection [5,6]. In a systematic review, disturbance in personal relationships was the main type of social harm in all phases [1]. Other studies address positive social outcomes of participating in HIV vaccine trials such as less risky sexual behaviour among trial participants [4,7]. These studies are, however, from countries where several HIV vaccine trials are being conducted. Little is known about experiences of volunteers in low income countries where few HIV vaccine trials have been conducted [8].

Tanzania is among low income countries involved in conducting phase I/II HIV vaccine trials [8]. One of such trials is HIV Vaccine Immunogenicity Study (HIVIS03), which was conducted among healthy volunteers in Dar es Salaam. Evaluation of immunogenicity test results from the volunteers indicated higher and broader immune responses [9]. It is felt that evaluating social and behavioural experiences of the volunteers during the trial may be a useful complement to immunogenicity findings. The social behavioural results may also be important in addressing various concerns at community level. Therefore, the purpose of this study was to evaluate what characterized experiences of volunteers during the HIVIS03 trial.

The interpretive description (ID) approach [10] was used to analyze the result data. By using ID, we provide contextual understanding to guide future research on people. ID generates new insights on what may characterize the experiences of individuals who enrol in HIV vaccine trials in a similar context.

## Methods

### Study setting

The clinical part of the HIVIS03 was conducted at a trial site located at Muhimbili National Hospital (MNH), Dar es Salaam, Tanzania. The trial was a collaboration between, among others, the Muhimbili University of Health and Allied Sciences (MUHAS) in Tanzania and the Karolinska Institutet in Sweden. This qualitative socio-behavioural evaluation study was conducted at the clinical trial site.

### Study population

The HIVIS03 project enrolled 60 volunteers (45 men and 15 women) in the Phase I/II HIV vaccine trial. All volunteers were recruited from the police force and all of them were police officers [11,12]. Before the vaccinations, potential volunteers attended a series of workshops and discussions with trial staff [12]. During these workshops, all participants were given written documents with details about the trial design, and the trial team encouraged them to share such documents with their relatives, friends and colleagues. Among other issues, the focus of these workshops was to discuss the criteria for enrolment in the HIV vaccine trial. The criteria included: be between 18 and 40 years old; be willing to stay in the study for the stated period of 24 months; women must not be pregnant and must use an effective contraceptive method for the duration of the trial; agree to HIV testing and adhere to condom use to prevent HIV acquisition, sexually transmitted infections and to avoid pregnancy. In the workshops, the trial team strived to provide precise and accurate information and answers to questions to the potential volunteers.

The implementation of the trial adhered to study protocol. After enrolling in the trial, a total of 16 study visits were spread over the trial period. During these visits, volunteers were screened through clinical history and examination [13]. Follow-up visits at the trial site were carried in accordance with the AIDS Vaccine Literacy Toolkit [14]. Volunteers received clinical care at the trial clinic for minor ailments, while for other ailments, they were referred to specialized facilities and such services were paid for by the trial insurance company. Before participation in the trial, participants were paying out of pocket and reimbursed by their employer in order to access medical services from public or private health care facilities.

At regular intervals during the trial, workshops were held to update the volunteers on the progress of the trial. During these workshops, volunteers were reminded about the importance of adhering to safer sex practices because the vaccine used in the trial was yet to be proven to protect against HIV transmission. The likelihood of possible false positive HIV diagnostic test results with standard HIV diagnostic antibody assays resulting from the candidate vaccines was impressed upon the volunteers. It was also emphasized repeatedly that the candidate vaccine products used in humans could never cause HIV infection in the vaccinees. Volunteers were advised that in case they required an HIV test in the future, the trial clinic would help them to have an HIV DNA PCR test done in the study laboratory which would distinguish between vaccine-induced antibodies and the presence of HIV DNA due to true HIV infection. Thus, during the trial, volunteers were advised not to have an HIV test outside the trial clinic. In each workshop, volunteers were invited to discuss various trial related issues.

### Study design

This was a qualitative evaluation study, nested in the HIVIS03 trial to evaluate social issues as stated in the project protocol. The qualitative method was chosen because it explores the shared perspectives and range of issues as expressed in the participants' own words better than the quantitative method.

### Recruitment

We made two announcements about this study during the regular workshops with the HIVIS03 volunteers. Firstly, we told them that they would be invited to focus group discussions to share their experiences during the trial after scheduled vaccinations, and we informed them that participation was voluntary. The second announcement was made in a workshop two months before some of the volunteers completed the last vaccination (MVA Boost) informing them that only those who had not participated in an earlier interview [15] would be eligible for the focus group discussions. Those volunteers who were eligible and willing to participate in the study were asked to confirm their participation by ticking their names in a pre-prepared list. All eligible potential participants who were in the workshop ticked their names on the list. Before the study, the first author grouped the potential participants according to gender and marital status. The number of participants per group followed the recommended size for focus group of 6-12 participants [16]. Thereafter, each participant was sent a letter of invitation to participate in the study on a specified date and time. On arrival at the study site, each participant completed a registration form with personal socio-demographic characteristics and was assigned a tag number (1-11) for anonymity during the discussion. An assistant [not the trial staff] assisted with the registration process.

### Sampling procedure and framework

**Figure 1:**
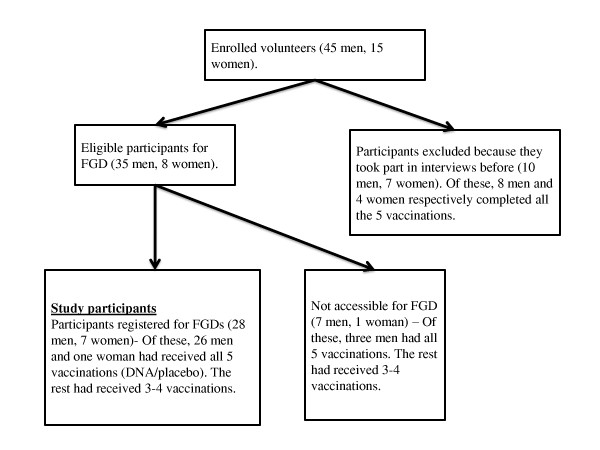
**Sampling framework**.

The study included a purposive sample of volunteers who participated in HIVIS03 trial, who were still in contact with the trial team, and were available during the study. Of the 60 trial participants, 17 participants were excluded from the sampling because they had shared their experiences in a previous interview [15]. Eight participants were eligible for the study but were not available during the last workshop when confirmation to participate in the discussions took place; they were noted that they were not in Dar es Salaam and they would be out of Dar es Salaam at the time of the planned study. The rest confirmed their participation and were invited to participate in the study (Figure 1).

### Data collection

We collected data between March and April 2009, after completion of two years of HIV vaccine trial study. Data was collected in a spacious room with chairs arranged in semi-circle for face-to-face interaction. We used focus group discussions (FGDs) because FGDs are typically designed to elicit normative feelings, responses, experiences, or world-views [17], and to meet time and resource constrains. Participants were arranged in groups (Table 1). A topic guide, which was prepared in English, translated into Kiswahili and translated back into English, was used to moderate the discussion. Each group was re-introduced to the purpose of the study, and given rules such as showing respect for each other, turning off mobile phones and the importance of identifying each other by number throughout the discussion. They were also reminded to share both positive and negative opinions, and that the information generated would be treated as group opinions or views. One question was asked: "Can you tell us your opinion on changes that occurred during the vaccination period?" This question was followed by probing questions. Throughout the discussions, participants identified and addressed each other by numbers tagged on their tops/shirts. The first author moderated all the discussions and a note taker [two academic colleagues interchangeably] took notes. The discussion was audio-recorded. Participants were served with a bottle of water during the discussion; soft drinks and snacks were served at the end of the session. They were also reimbursed in Tanzanian shillings [equivalent to $ 20 USD for transport to and from the study site]. The discussion sessions lasted between 79 and 115 minutes.

**Table 1:** Participants

Group	Participants' characteristics	Number of participants
1	Men (married [5] and unmarried [4]), mean age 27 years old	9
2	Men (all unmarried men), mean age 28 years old	11
3	Women (married [2] and unmarried [5]), mean age 27 years old	7
4	Men (all married), mean age 36 years	8
**Total**		35

### Data analysis

Interpretive description (ID) approach informed by principles of thematic content analysis was followed when analyzing the data [10,18]. Analysis began immediately after each FGD. The first author listened to the audio-recorded material and compared the identity of each participant with the note-taker's summary for consistency. This process allowed the author to understand the clarity of voices in the audio-recording and define key phrases that were specific to certain people in the setting of the study [19] One assistant transcribed the audio-recorded material; another translated the transcripts from Kiswahili into English. For consistency, both versions were read and checked by two authors, EAMT and TWK who speak Kiswahili and English. Differences in language interpretation were discussed and both Kiswahili and English quotes were re-checked for consistency. EAMT matched each transcript with the note-takers' summary [key point of the speech and identity of the speaker] and the registration form. This matching enabled the author to merge individual tag number [identity], marital status and group number in each transcript for quotations.

**Figure 2:**
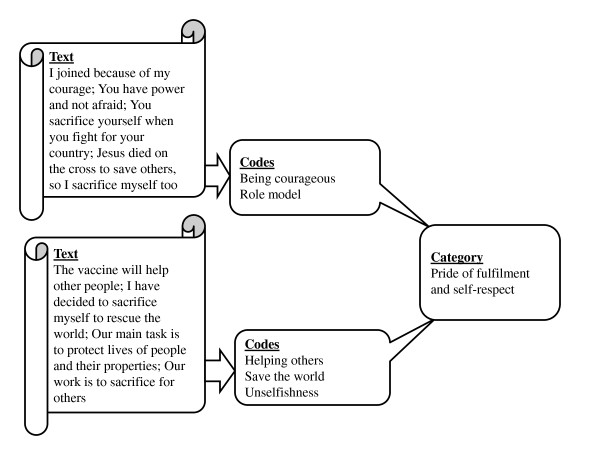
**Example of coding process**.

In the process of interpreting the findings, EAMT and TWK coded each transcript paragraph by paragraph. The manual coding carried the participant identity [sex, tag number, and marital status] and group number as text locators. The process of coding was inductive in nature. By using an inductive approach, we sought to explore the data and to identify emerging findings. The initial phrase consisted of identifying codes and giving them a distinct code. Further, EAMT and AK tested the coding by trying different angles of vision, while appreciating the implications of each option for grouping and reconstructing the categories. The initial inductive coding was aimed at primarily identifying descriptive codes. This was followed by the process of bringing together all codes which were related. Following this process, categories were formed from the codes (Example given in Figure 2). In the process of moving from codes to categories, an attempt at interpretive analysis was included. Data within the data set were systematically compared to ensure no key finding was left out. Following the identification of categories, these were brought together into two themes. The themes emerged from the underlying meaning of the categories while they also stayed close to application potentials of interpretive description [20]. Our findings were cross-checked with trial participants in order to increase trustworthiness. Participants consented to the interpretation made.

### Ethical considerations

The HIVIS03 trial project to which this evaluation study is affiliated was approved by the National Ethics Committee at the National Institute for Medical Research (NIMR) which offered a letter with reference number NIMR/HQ/R.8a/Vol.IX/410. A thorough process of obtaining informed consent and satisfying eligibility criteria was a prerequisite for enrolment in the trial. Before the discussions, potential participants gave verbal consent after re-describing the purpose of the study. They were reminded about the issues of confidentiality and their rights as participants. They were also asked to adhere to the principles of shared confidentiality [between the group members and the researcher], and they were asked for permission to tape record the discussion. They all gave their consent and participated fully in the study.

## Results

### Participants

Of the 35 participants, seven were women and the age ranged between 23 and 42 years. A total of 13 men were married and 15 were single. Two and five women were married and single respectively (Table 1). Although we did not explore about the participants HIV risk factors such as homosexuality or heterosexuality, those who were in sexual relationships indicated that they had partners or spouses from the opposite sex. Twenty seven participants had four years of secondary education and above. The rest had seven years of primary education.

### Themes

Two major themes emerged from the analysis, 'Embracing benefits of participating in an HIV vaccine trial' and 'Encountering and handling mistrust about an HIV vaccine trial'. Within each theme, four categories were formed (Table 2).

**Table 2:** Themes and categories

Themes	Categories
Embracing benefits of participating in an HIV vaccine trial	Reducing risky sexual behaviour Overcoming fear of HIV testing
Encountering and handling mistrust in an HIV vaccine trial	Privilege of complete medical check-ups Changed to better health-seeking behaviour
	Dealing with attitude of mistrust of non-trial health care providers Confronting discouragement from colleagues Convincing the family Pride of fulfilment and self-respect

### Embracing benefits of participating in an HIV vaccine trial

#### Reducing risky sexual behaviour

Participation in the HIV vaccine trial was experienced as beneficial to the participants' sexual life. Before the trial, the participants confessed that they were poorly informed about HIV transmission. They stated that the information in the trial enabled them to change their behaviour and practice safer sex. In other words, they became optimistic about safer sexual lifestyles. The male participants said they decreased the number of sexual partners as a result of the information from the trial. They asserted that although risky sexual behaviour was part of their daily lives, they managed to adopt safer sexual practices due to the information in the trial. Other participants asserted that they changed their behaviour at different points along the trial. Some of them changed after a negative HIV test; others were encouraged to maintain positive behaviour after the second and the third DNA injections, something which is exemplified by the following quote:

"For my part, I changed the first day I tested. I think that the test made me change, and the results were clean. Truly, I gained courage, and I was very happy... That has helped me a lot to change my behaviour." (Man 3, married, Group 1)

Besides changing risky sexual behaviour, participants also reported that they advised others to practice safer sex:

"Those seminars helped us a lot because from the first vaccination, we were told that wearing a condom is really important. That one helped because some of us had multiple sexual partners... It [seminar] has helped and strengthened me a lot. I mean, I do not have strange things [risk behaviour].... To a large extent, I was able to advise my friends." (Man 6, married, Group 1)

Women stated that they benefited from the correct information on how to avoid pregnancy and sexually transmitted infections. In addition, the participants believed that the routine HIV testing in the trial contributed to the behaviour change:

"You find testing for HIV is voluntary, but when you are in the research [trial], that voluntarism is not there. So it compels you to protect yourself in any way. Now you find yourself automatically changing your behaviour. It means you are forced to live a certain lifestyle..." (Man 3, unmarried, Group 2)

Acceptability of condom use emerged as an engaging topic for discussion among the participants, who claimed that the information in the trial helped them to understand the value of using a condom to prevent infections and pregnancy. Although other participants were confused whether to consistently use condoms with their stable sexual partners, most of them declared that regular seminars strengthened their trust of condom use, and some admitted they were now being more careful during intercourse even though they were wearing a condom.

#### Overcoming fear of HIV testing

The participants said that the fear associated with testing for HIV status had been eliminated by the trial interventions. Participants declared that they had more confidence in HIV testing compared to their colleagues who were not in the trial:

"After joining this research, I have gained confidence which I did not have before. That is confidence to test for HIV status, readiness to take whatever test results, something that would be very difficult for other people." (Man 3, unmarried, Group 2)

Also, participants added that during the trial they felt more confident because of the results from HIV testing and reflections on their health status:

"When you look at your [my] own health, and it is progressing well and telling a person that, 'I checked my health status yesterday and I have no HIV infection and my results are here', he/she understands even though I am enrolled in the HIV vaccine trial." (Woman 7, married, Group 3)

Most of the participants said that the issue of HIV testing did not trouble them after enrolling in the trial. They emphasized that they were very careful to protect themselves against HIV transmission.

#### Privilege of complete medical examination

Enrolling in the HIV vaccine trial was perceived as a privilege in accessing medical services. For instance, participants experienced the trial as a way of being able to have voluntary medical check-ups. Through these check-ups, most of them said that they learnt about their general health status. They appreciated this opportunity because before the trial most of them did not bother to seek professional medical advice. In the trial, they adopted the habit of checking their health status even when they were not sick. The group participants emphasized the same message:

"Even me, I benefited... The first benefit we got is check up; I know my health status on what is going on..."

According to the participants' past experiences, medical check-up was not a priority. They emphasized that they only sought medical check-up when it was necessary. Thus, courage and hope after being properly examined and receiving medical treatment emerged as an important benefit of enrolling in the HIV vaccine trial:

"Everything you checked, you were told by the doctor, that you were okay ... when you went out, you got a relief that you did not have any problem. You see, because if a person had kidney problems, he was given treatment. Therefore, that gave us courage and hope..." (Man 6, married, Group 4)

#### Changed to better health-seeking behaviour

Before enrolling in the trial, the participants said that they used self- medication. During the trial, however, they accessed professional services through counselling, consultation; proper treatment and regular follow-up. They learnt about the importance of adhering to professional advice throughout:

"Being in the research has influenced me to leave the doctor free to check my health status and decide which medicine to use." (Man 7, married, Group 1)

They said that the tradition of self-prescription was enhanced by perceived lack of trust in doctors:

"Previously, I feared to consult the doctors, and I didn't like it. I perceived them differently. When I fell sick, I bought my Panadol and took it. That is it..." (Man 3, married, Group 4)

Possible causes of hesitations to consult doctors in time of illnesses were aired by other participants. They felt that doctors investigated more than what the clients expected from them:

"First of all you may go to consult a doctor because of 'malaria', but through his professional expertise, he may ask you to give blood from this site [pointing at a common site where blood is taken for tests like HIV]. I think you will even escape to pick the medications, and you will not return for the test results.... This is because of the existing problem [HIV]." (Man 4, married, Group 4)

Overall, participants experienced the trial follow-ups as a way of accessing professional health services which promoted confidence in their HIV status, general health status, and in their relationship with the trial team.

### Encountering and handling mistrust about the trial

#### Dealing with the attitude of mistrust of non-trial health care providers

Despite the opportunities in the trial, the participants faced challenges from health care providers who were not connected to the trial. The participants were unexpectedly forced to interact with health care providers who were not informed or poorly informed about the HIV vaccine trial. Interaction with these providers was difficult. Although they were surprised by the attitude of mistrust among these providers, they managed to use active and passive strategies to defend their decision to enrol in the trial. They shared experiences and how they reacted when they interacted with medical doctors [doctors who were not connected to the trial] facilitating an HIV and AIDS workshop at their workplace as exemplified in the quote:

"This program [HIV vaccine trial] is in the police force, but I feel very sorry for those who joined in because they will be seriously affected' [said a doctor]. After hearing that, I neither responded nor showed up that, I was among the trial participants! I took him as he was." (Man 3, unmarried, Group 2)

This attitude of mistrust was not just among medical specialists. The participants also noted opposition to the HIV vaccine trial from other health care providers. Another participant shared his encounter with a laboratory technologist:

"I can add on that ... it is the same people; those whom we call health experts and who are not in the trial. Somebody who is a laboratory technician... Intentionally, you could see him/her mobilizing people and being the main speaker, misleading them about the trial... Being part of it [trial volunteer], you keep quiet; pretending not knowing what he is talking about ..." (Man 4, unmarried, Group 2).

The participants were confused when faced with mistrust among medical doctors over the trial, especially when they sought medical consultation outside the trial clinic. Despite their expectations that all health care providers would know about the trial, surprisingly, they realised that most of them did not know or were poorly informed. Under such circumstances, the participants stated that they were confused:

"When you tell a person that you are involved in something [HIV vaccine trial], and he/she comments 'he!', in your heart you feel: 'Does it mean that I am lost?'... I met a specialist, the one they call an orthopaedic surgeon. Just by seeing my documents [HIV vaccine trial volunteer], he was shocked in such a way that shocked me too ... then he called his nurse; she also looked at the documents in brief, and then they looked at each other. Things like those, we just say, let us go ahead..." (Man 9, unmarried, Group 2)

Older married men encountered a similar situation and they were also confused by negative comments from health care providers. One participant expressed his feelings after interacting with a medical doctor who was poorly informed about the trial, but he passively managed the situation. He said:

"This thing [vaccine trial] is impossible. Personally, I know the side effects, those of you who joined are insane' [A doctor commented]. These are truly health care experts of whom we expect to be champion in such issues, but they are the main barriers... we just volunteered because of courage... If I die, it is okay, and if I survive 'Insha'Allah', you see? But we were disturbed." (Man 6, married, Group 4)

Generally, the participants expressed disappointment following the reactions of the health care providers outside the trial team.

#### Confronting discouragement from colleagues

Regarding existing relationships and daily interactions at the workplace, participants' enrolment in the HIV vaccine trial was something that distinguished them from their peers. The participants stated that their time spent at the workplace became less enjoyable during the trial. They said they met discouragement from colleagues in the form of jokes, and rumours that the vaccine trial was not safe. They also noted that the discouragement was frequently accompanied by negative opinions towards them. Sometimes they felt discriminated against, humiliated and upset when colleagues and friends commented negatively about them. They either actively educated them, confirmed that they were HIV negative or passively kept quiet to maintain a peaceful situation. One participant said:

"At our workplace, people joked a lot: 'You have been transplanted with HIV' [colleagues said]. Sometimes it discouraged us; you find yourself arguing with people ...You find somebody scorning you; you just ignore him/her. At most I would call him/her and educate even if he/she did not understand." (Man 6, married, Group 1)

Other participants experienced disturbance when colleagues who were not in the trial stigmatized them in front of other people. One man said a colleague pointed his finger at him in a canteen in front of more than twenty people, 'This is the one who has been transplanted with HIV virus.' [He shouted]. He said the situation forced him to defend that he was only taking part in the HIV vaccine research, not transplanted with HIV, but they [civilians] continued to question him. To clear the doubt he disclosed that he had re-tested and he had confirmed that he was not infected with HIV. Also, other participants added that they had repeated HIV testing in different places to confirm that the vaccine was not infectious:

"I have talked to that one [colleague] for a long time to clarify the truth, and I told him that besides the tests at the trial site, I have been re-tested to see whether I am infected or otherwise. I have tested in different places where they do not even know me in order to gain more confidence." (Man 7, married, Group 1)

Commonly, participants faced difficulty in clarifying the information about the vaccine trial to people because of the novelty of it among community members. They thought they could give up, but they realized that they should continue to educate those who were ready to pay attention:

"We are facing difficulties in educating a person who does not know it [HIV vaccine trial] and make him/her understand. Although in the place where we live, there are people who talk nonsense in front of others, we need to accept them, they are our colleagues, and we cannot avoid them." (Man 4, unmarried, Group 1)

Although the participants stated that the workshops strengthened their knowledge about the vaccine and its safety, they still experienced difficulties in delivering the information to the community. Women felt that the friends believed that the HIV vaccine trial had negative consequences on their reproductive health:

"And another person suspected that I could not give birth because of the vaccine. I mean for example at the workplace, aah, truly God helped us. We have come a long way and now we are breathing... They saw us as people who are infected with the virus...waiting to die and that confused us." (Woman 1, married, Group 3)

Other participants recognized that their colleagues were making signs of contempt behind their back when they passed, inferring that they were already infected. Although they realized that the negative opinions they came across were linked to mistrust of the HIV vaccine in the community, they felt traumatized with such opinions especially during illness episodes. One participant said her boss disappointed at her bedside in the hospital to which she had been admitted for a surgical procedure [non-trial-related]. She said that her boss offended her by saying, 'those people [researchers] are selling you, and they benefit because of you [said the boss]'. She felt disappointed and placed everything on faith. She lamented:

"Given that I was sick, then hearing such words! The only answer I had was: 'If dying is for human beings, living is also for human beings... In the beginning, you take those words, but often they are painful!" (Woman 4, unmarried, Group 3)

Another woman related her experience during illness and how she passively reacted while interacting with colleagues:

"I was suffering from typhoid illness...When I went to the office they [colleagues] told me that, 'You know it is that vaccine that brings such illness'. I remained quiet and asked myself, 'don't they even care that I am sick? To tell the truth those words were painful..." (Woman 6, unmarried, Group 3)

The participants continued to struggle against people who were discouraging them. Although, they realized that some were labelling them as 'people who are confused by enrolling in the trial', they believed such negative views could not change their mind set because of the correct information they had received on the trial.

#### Convincing the family

The common comfort zone, the family where supportive networks are established, turned out to be a discomfort zone during the trial. Several participants noted mistrust of the trial among members of the family when they said they were participating. Discomfort often emerged when close family members, such as parents, sexual partners, and blood relatives, demonstrated mistrust of the vaccine safety. Under such situations, participants were forced to carefully deal with the doubts to maintain existing relationships. For instance, some men reported that their wives were distressed by the comments from the surrounding communities that their husbands were taking part in a harmful study. Under such a circumstance, they tirelessly educated them. Other participants admitted that their pre-understanding about their families was helpful to deal with the situation. Some of them opted to share the information with their intimate friends, but not their mothers because of perceived different levels of understanding:

"I tried hard to explain to my lover but when it came to the other side, the side of the parents... I didn't tell my mother this issue because I knew it would take time for her to understand..." (Man 3, unmarried, Group 2)

On the contrary, those who shared their enrolment in the trial with relatives, they felt abandoned. However, they passively kept quiet and continued with the trial. One man shared his experience:

"I tried to explain [about the trial] to my relatives. Truly, all of them threw me out of the line [discouraged me]... So, I am alone; they find its okay. When I phoned and told them that I have fever, they say: 'You wait, go ahead!' [The relatives warned]. So those are their current responses. Now even if I have mild-fever, I don't call them." (Man 7, unmarried, Group 2)

Other participants in the group sensed that the reaction from the family members was more negative than that of friends. For example, they noted that some parents were shocked after realizing that, without their knowledge, their sons and daughters were participating in the HIV vaccine trial. One woman shared how her father was shocked and how he forced her to re-test for HIV to rule out any negative consequences of taking the vaccine. After the first two DNAs, she said her father pretended to organize a family event that every family member had to accompany him on the AIDS day, December 1^st^, to have an HIV test. Thus, the whole family went with him to the HIV testing centre [not at the trial site]. On the contrary, some of the participants experienced comfort from their families. To some extent, they sensed good relationships. Nevertheless, one participant pointed out that the relationship was maintained because the vaccine had no visible side effects.

Therefore, some participants had positive interaction with their families during the trial while others did not. On top of looking healthier, they relied on their individual decision that pushed them to enrol and eventually stayed on until the end of the trial. The following section highlights these individual motives.

#### Pride of fulfilment and self-respect

Participants felt they had made an important decision and believed that self-sacrifice, self-courage and faith in the vaccine trial were important to accomplish their contribution in the HIV vaccine development. Notwithstanding the discouragement they received, they realized that individual decision was crucial:

"It [discouragement] is there. Others were pressing me until I decided to tell them, 'I have decided to sacrifice myself; I have already sacrificed to rescue this world. If it is a vaccine, then it will help other people... Jesus died on the cross to save others. So, I sacrifice myself too ..." (Man 7, married, Group 4)

Through body gestures, other group participants indicated that this was an experience they could identify with. Most of them reflected on a similar situation when they interacted with colleagues and friends. The participants referred to their core role as an opportunity to protect people and their properties. Thus through participation in the trial, they believed that they would also protect people's health. They emphasized that taking part in the trial was a courageous act to save the nation from the HIV calamity:

"You have power and are not afraid. You sacrifice yourself when you fight for your country ..." (Woman 6, unmarried, Group 3)

Individual decision was the basis of volunteering for the HIV vaccine trial. Thus, to a large extent, the participants managed to deal with the doubts in the trial because of this decision. They also felt they were able to ignore negative comments from others because of confidence they had in the trial from the start, and asserted that participation in the trial was a personal decision sometimes driven by the death of a relative from AIDS:

"To me it is my own decision. When I decide to do something and I see it is beneficial to me, I just do it ...we have lost two relatives [AIDS-related death] from our family. Truly, it hurts me so much. So, when I heard about this vaccine trial, I decided straight away. I mean, nothing can change me." (Man 6, married, Group 1).

Also, they felt confident because of regular contacts with the trial team. They said they experienced warmth and friendliness during such contacts. They boasted that they knew more facts about HIV and vaccine than their colleagues because they attended the workshops.

## Discussion

This socio-behavioural evaluation study shows that during the phase I/II HIV vaccine trial in Dar es Salaam, the participants' lives were characterized with both positive and negative experiences. The trial interventions promoted individual gains in terms of safer sex, habit of having HIV tests when ever need arose, access to free (insured) medical services during the trial period and establishment of good relationship with the trial health care and research team. In spite of these opportunities, the participants faced several challenges when they interacted with people who were not connected to the HIV vaccine trial research. The volunteers actively or passively managed to cope with the doubts, stigmatisation, and misinformation throughout the trial period. They demonstrated pride of fulfilment and self-respect when committing to their participation in the trial.

The experience of change from risky to safer sexual behaviour of the trial participants implies individual gain through the trial interventions. The regular monitoring of HIV status and counselling may have greatly contributed to the positive change in behaviour. However, the big challenge is whether or not the volunteers sustain the safer sexual practices in absence of the trial interventions. A previous study on this trial population showed that participants' willingness to participate in an HIV vaccine trial was associated with perceived risk of getting HIV infection [11]. Elsewhere, participants in efficacy trials engaged in risky behaviours consistently over the 6-, 12- and 18-months follow up visits [21], but a decrease in risky behaviour in phases II [7] and III trials has been documented from other studies [22-24]. However, it is not predictable which trend the post-HIVIS03 volunteers will follow given the differences in context and type of the HIV vaccine trial they were enrolled in and the environment they will continue to be in. While the volunteers in the HIVIS03 were generally perceived to be at a low risk of becoming HIV infected at the time of enrolment, the findings in this sub study show that several of them perceived themselves at risk. In Tanzania, risky sexual practices and not having regular HIV tests overrides the need for the national advocacy on behaviour change, counselling and voluntary HIV testing among other appropriate interventions [25]. The current findings however, signify the contribution of the trial interventions to the behaviour changes; changes that seem glowing according to the participants' own expressions. While the appreciation by the volunteers for the HIV tests and general medical check-ups received during the trial contradicts with the earlier findings from the similar population [12], we believe that access to precise information, health services and the availability of medical insurance in the trial are the core sources of the appreciation. The experience of volunteers gained through the trial interventions supports the findings of others [3,7,23].

The mistrust of significant others on the HIV vaccine trial is not surprising. In the similar population, significant others were potential barriers [12] and the main reason for declining to enrol in the HIV vaccine trial [13]. What is now becoming serious is the attitude of mistrust of the health care providers who, because of their professional background, would be expected to support the medical interventions. This situation demands a wider approach for dissemination of information on HIV vaccine trials, particularly to specific groups of stakeholders in the communities from which trial participants are drawn. The Good Participatory Practice (GPP) guidelines for biomedical HIV prevention trials provide a list of stakeholders which include trial participants; families of trial participants; community members residing within or in the surroundings; the research catchment area; people living with AIDS or affected by HIV; advocates and activists; non-governmental organisations; community based organisations; religious leaders; opinion leaders; media; government bodies; national and local health care authorities; service providers; trial funders; trial sponsors and trial implementers [26]. Also, Allen and Lau provide a model to prevent social impact related to HIV vaccine trial participation [27]. Although the mistrust of significant others (not medically trained) about HIV vaccine trials has been documented in previous studies [2,3,5], the utilisation of the above guidelines will substantially increase the dissemination of trial information.

The strategies employed to cope with the doubts in the HIVIS 03 trial may have been greatly enhanced by the routine follow-ups, services and the precise information about the phase I/II trial. However, some volunteers underwent HIV tests outside the trial clinic because of the confusing rumours from the surrounding community. The negative reactions of the colleagues towards them reflect the misconceptions about the vaccine in the surrounding community. The participants' response regarding how they coped signifies that human beings are passive recipients of social environment influences to which they simply respond [28]. While interacting with non-trial health care providers, participants simply behaved neutrally implying that they avoided arguing with the 'perceived knowledgeable' people. This could be based on the way in which they relate to actions [28]. Nevertheless, at some points they were overwhelmed beyond their ability to accommodate the instructions from the trial staff. Here, the participants were obliged to defend the trial safety, not only by providing correct information about the trial, but also by quoting evidence from outside the trial site. Nevertheless, the participants maintained trust in the trial until the end, mostly because of the trial interventions.

### Methodological considerations

This qualitative study was planned to cover the first part of the vaccination period, stretching from the first to the fifth vaccination. The second part of the trial was unblinded and volunteers told whether they had received an active vaccine or placebo. We note that unblinding occurred after data collection for this study, and all volunteers who had received all the five active vaccine products exhibited vaccine-induced HIV-1 seropositivity [29]. It would be of great interest to have an idea on how those who tested positive for HIV antibodies but were HIV DNA negative behaved or responded socially, and how their significant others reacted (for those who got informed). However, the experience gained from the first part of the study is broader and useful for future trial implementers. Understanding of socio-behavioural reactions of the volunteers and their significant others after unblinding will perhaps increase our knowledge as regards phase I/II HIV vaccine trial participation.

Although we cannot generalize these findings beyond the studied sample of volunteers, the information obtained sounds crucial when it comes to designing HIV vaccine trials in similar contexts. The use of qualitative approach allowed us to explore a scientific area that has not been well investigated before. Even though the use of FGDs could have limited the discussion to sensitive sexual matters [30], they provide findings which can often be used as a basis for actions [31]. Similarly, by using ID approach, data analysis is oriented towards the development of useful findings for practice [32]. Intentionally, in this evaluation, we excluded the participants who shared their experiences in interview right after the third DNA or placebo injections. This allowed the researchers to gain collective experiences from those who had no chance to share them. The moderator of the FGDs being part of the trial team may have influenced the participants not to disclose negative experiences gained at the trial clinic. However, we believe that the trial staff, with whom the participants had regular contact may have taken care of any immediate concerns they may have had.

In terms of reflexivity, participants demonstrated trust in the moderator [EAMT] of FGDs possibly because of her presence in the workshops before and during the trial. In spite of introducing her role as a social scientist in the trial project, the participants perceived her as a representative from the trial team. While this seemingly did not pose a problem, it is impossible to say whether this misinformation led to a censuring of some content. However, since both positive and negative views were expressed on most topics discussed, we see this effect as minimal.

### Implications

In future trials, it is crucial to conduct quantitative longitudinal studies to monitor possible post-trial behaviour change. The mistrust that we witnessed in this study can be minimized by timely dissemination of the trial information to the respective stakeholders. Involving past trial volunteers in the sensitization or recruitment meetings of prospective future vaccinees may provide valuable evidence on trial safety to the surrounding community. Regular follow-ups can build and sustain good rapport between the volunteers and the trial staff. In particular, when the trial volunteers encounter doubts from outside the trial, they should continue to have access to immediate support or someone to turn to for reassurance. The proposed guidelines of good participatory practice in conducting biomedical trials need to be implemented and monitored according to the trial context. The most important group to start with would be the health care providers in the respective community; they are the most trustful people when it comes to health-related information. Also, the findings suggest that volunteers who decide to enrol in the HIV vaccine trial do show a degree of independent thinking when resisting discouraging messages.

## Conclusions

The article presents an evaluation of the volunteers' experience in an HIV vaccine trial in Dar es salaam, Tanzania. The benefits experienced by the trial participants are connected to the interventions in the trial such as regular follow-ups, medical check-ups, counselling and regular interaction with the trial team. Such benefits are however tempered by the challenges from the surrounding community. The issue of mistrust of the trial from health care providers who were not connected to the trial is more serious than the discouragement from the colleagues. However, personal commitment and trial-related interventions were important for the participants to stay on in the trial.

## Competing interests

The authors declare that they have no competing interests.

## Authors' contributions

EAMT conceived the study, coordinated data collection, carried out analysis and drafted the manuscript. AK and TWK participated in the study design, data analysis and critically reviewed the final draft. AT, MB, ES and FM were involved in study design and critically reviewed the manuscript. All authors read and approved the final manuscript.

## Pre-publication history

The pre-publication history for this paper can be accessed here:

http://www.biomedcentral.com/1471-2334/11/283/prepub
